# Rivers constrain female but not male dispersal and genetic structure in brown bears

**DOI:** 10.1038/s41598-026-38870-4

**Published:** 2026-02-09

**Authors:** Robert Spitzer, Anita J. Norman, Michael Schneider, Ellinor Sahlén, Göran Spong

**Affiliations:** 1 Department of Wildlife, Fish, and Environmental Studies, Swedish University of Agricultural Sciences, Umeå, Sweden; 2 Västerbotten County Administration, Umeå, Sweden

**Keywords:** *Ursus arctos*, Dispersal, Non-invasive genetic sampling, SNP, Conservation genomics, Behavioural ecology, Population genetics, Conservation genomics

## Abstract

Understanding landscape barriers to connectivity is essential for studying wildlife population dynamics and developing conservation strategies that promote genetic exchange. Rivers can fragment landscapes and thereby influence genetic metapopulation structure by restricting individual movement and gene flow, yet their impact on brown bear (*Ursus arctos*) dispersal remains poorly understood. Using a large dataset (N = 519) of SNP-genotypes (96 loci) from fecal samples, we investigated the effects of rivers on sex-specific movement patterns, primarily dispersal and genetic structure in brown bears in northern Sweden. We found that males dispersed over twice as far as females (mean 56.4 km vs. 22.8 km) and crossed rivers significantly more often (42% of male vs. 11% of female dispersals; χ^2^ = 49.33, *p* < 0.001). Simulated female dispersals in random directions showed higher river crossings (17.7%, t = 90.47, *p* < 0.001), suggesting philopatry alone cannot explain the low observed crossing rate. Rivers thus acted as semipermeable barriers for females, with genetic structure analysis (DAPC, spatial PCA) showing weak structuring effects for females, but none for males. Our study underscores the need to identify specific crossing sites, evaluate additive effects of other barriers (e.g., roads), and expand research across Sweden to guide connectivity-focused conservation.

**Supplementary Information:**

The online version contains supplementary material available at 10.1038/s41598-026-38870-4.

## Introduction

As populations of large, widely roaming carnivores experience increasing human presence in the landscape, causing habitat fragmentation and smaller population sizes, maintaining connectivity is important for preserving genetic diversity and demographic processes. Connectivity plays a vital role in supporting wildlife populations by ensuring gene flow, which helps maintain genetic diversity—a key factor for species to adapt to ecological challenges such as changing environments or resisting disease. Without sufficient connectivity, populations become isolated, gene flow decreases, and the risks of inbreeding, reduced fertility, and susceptibility to disease increase.

Furthermore, connectivity is essential for demographic resilience in several ways. Large carnivores require large territories to find food, mates, and suitable habitat. Connected populations allow individuals to move across landscapes, enabling them to escape areas with scarce resources or unsuitable conditions.

In addition, connectivity plays a key role in processes like recolonization or reintroduction, allowing species to return to areas where they were previously extirpated. Without these vital linkages, such processes are hindered, limiting a species’ ability to occupy new or restored habitats, and ultimately threatening their long-term survival.

The Scandinavian brown bear (*Ursus arctos*) has undergone a remarkable recovery over the past 100 years, with numbers rising from a hunting-induced bottleneck of approximately 130 individuals in the 1930s^1^ to a current (2023, after the hunt) population estimate of approximately 2500 in Sweden^2^ and 130 in Norway^3^. Genetic studies have identified three partially connected subpopulations of brown bears in Sweden^4,5^. These genetic differences largely reflect postglacial recolonization routes, with bears expanding into Scandinavia both from central Europe in the south and from eastern Europe and northwestern Eurasia in the north^6–9^. The two northern subpopulations descended from the eastern lineage, whereas the southern subpopulation originated from a western lineage that likely had its source in central Europe^6^ rather than the Iberian Peninsula, as previously assumed^7,8^.

Investigating potential barriers to connectivity is essential for understanding how brown bear (meta)populations interact across landscapes and for identifying conservation strategies that promote genetic exchange. In an increasingly fragmented landscape, the possible effect of rivers as barriers to movement could have important implications for metapopulation dynamics. However, little is known about the influence of major rivers on bear movements and dispersal. We have been unable to find any studies addressing this question for brown bear, but evidence from two studies on American black bear (*Ursus americanus*) suggests that such effects exist. Tracking the movements of 40 radio-collared black bears in Louisiana, White et al.^10^ found that a medium-sized river (~ 200 m wide) was crossed by 15 of 19 males (79%), but only 8 of 21 females (38%), and that none of the bears crossed the larger Mississippi River (~ 1600 m wide). Bond et al.^11^ were able to show similar, sex-specific effects of an even smaller river (~ 53 m wide) on black bear movements in Georgia; 7 of 17 (41%) collared males crossed the river compared to only 1 of 9 (11%) collared females. The possible explanations offered by both studies were that females have smaller home ranges and disperse over shorter distances than males and are thus less likely to be faced with the need of crossing rivers to establish territories. Secondly, females maximize their fitness through investment in parental care^12^. The presence of young cubs with limited vagility may therefore preclude successfully breeding females from crossing rivers due to the associated risks (e.g. drowning or separation from their mother by currents) to their offspring.

**Fig. 1:**
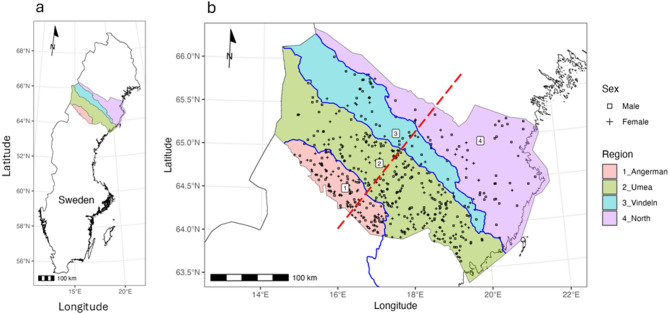
(**a**) Map of Sweden showing the location of the study area Västerbotten county. (**b**) The study area divided into four regions (colored and numbered) demarcated by three major rivers from south to north: 1—Ångerman River, 2—Ume River, and 3—Vindel River. The dashed red line indicates the reference line for dispersal angles (orthogonal to rivers; see Methods). Locations of individual brown bears (N = 519) are shown based on the center coordinates of hunter-submitted fecal samples, with squares representing males (n = 265) and crosses representing females (n = 254). The maps were generated using QGIS v3.36^16^ and R v4.4.2^17^ using administrative boundary data from Global Administrative Areas (GADM) v4.1 (https://gadm.org) and hydrography data from Lantmäteriet (Swedish national geodata), accessed under an SLU university employee license that permits use in academic publications.

Here, we use a panel of 96 SNPs developed for genotyping Scandinavian brown bear^5^ to test sex-specific effects of rivers on brown bear movement and genetic population substructuring on a large dataset of brown bears (N = 519 individuals) from the Swedish county of Västerbotten. Västerbotten offers ideal conditions because it harbors a large population of an estimated 513 (95% CI: 478 – 551) brown bears^13^ that is still expanding, and because the county is naturally divided by three major rivers (Ångerman, Ume, and Vindel Rivers, Supplementary Table S1) into four regions (Fig. 1). Genotyping from citizen-collected eDNA samples in the form of feces in our study, offers a cost-efficient approach for studying elusive species like brown bear over large spatial scales, but also has some limitations.

Being restricted to sample collection coordinates, our data lack the fine-scale spatiotemporal resolution typically achieved through telemetry studies and are missing some key demographic details such as age or breeding status. Although pedigree reconstruction to infer kinship between related individuals is possible without age information^14,15^, this approach remains uncertain and reduces the effective sample size. These limitations, however, did not impede our ability to address the primary research questions. We based our analyses on 1st-order relatedness (i.e., parent–offspring or full-sibling relationships), using male-male and female-female dyads as focal points. This approach is grounded in the assumption that such individuals are likely to have co-occurred spatially at some point in time. As a result, straight-line geographical distances between individuals in these dyads serve as a proxy for minimum dispersal distances^14^, providing a basis for assessing sex differences. In cases with large sample sizes, these distances are likely to reflect true dispersal differences between males and females. In this study we use the term ‘dispersal’ to describe the spatial distance between sampled 1st-order related individuals, which could have resulted from natal dispersal, but also other drivers of movement such as the search for mates, food, and exploratory excursions.

When evaluating rivers as potential barriers to dispersal, we were limited to determining whether any rivers were crossed by individuals in a dyad, but could not establish which specific individual undertook the crossing. However, this limitation did not undermine our capacity to detect potential sex-based differences in dispersal patterns. Specifically, we addressed three research questions by testing five associated hypotheses:

*Question 1: Do major rivers present sex-specific barriers to brown bear dispersal?*

### H1:

*Males are more likely to cross rivers than females.*

*Question 2: Do dispersal patterns between 1st order relatives differ for male and female brown bears?*

### H2:

*Dispersal distance for male-male and male–female pairs is larger than for female-female pairs.*

### H3:

*Dispersal direction in males is random, i.e. unaffected by rivers.*

### H4:

*Females are less likely to disperse orthogonal to rivers.*

*Question 3: Does genetic population structure of males and females reflect the geographic regions demarcated by major rivers?*

### H5:

*The effect of rivers on genetic population structure is stronger for females.*

## Results

The final dataset comprised 519 individuals (265 males and 254 females). Among first-order relatives, dispersal events were observed in 163 male-male dyads, 238 female-female dyads, and 362 male–female dyads.

### Males are more likely to cross rivers than females (hypothesis 1)

**Fig. 2:**
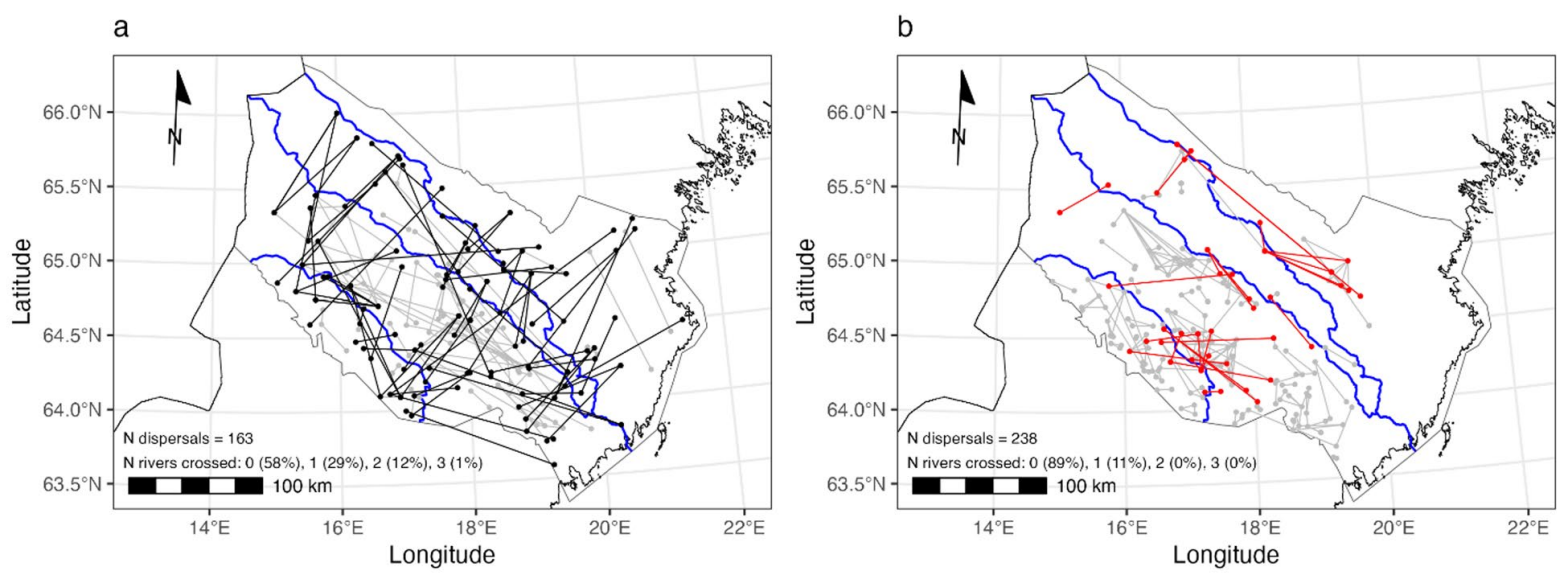
Brown bear dispersal events between (**a**) 1st order male relatives (N = 163) and (**b**) female relatives (N = 238). Dispersals that include river crossings are marked in black for males and in red for females; dispersal events without river crossings are shown in gray for both sexes. The proportions of dispersal events involving different numbers of river crossings are shown in the bottom left corner. For example, 29% of observed male-male dispersal events included one river crossing, compared to 11% among female-female dispersals.

We found strong support for our first hypothesis; 42% of male-male dispersals included at least one river crossing compared to only 11% of the female-female dispersals (χ^2^ = 49.33, *p* < 0.001; Fig. 2). Simulated female-female dispersals in random directions resulted in a higher proportion of river crossings (17.7%, t = 90.47, *p* < 0.001). The observed value for river crossings in female-female dispersal events (11%) fell outside the 97.5th percentile at the lower tail of the distribution for river crossings in simulated female-female dispersals (Supplementary Fig. S1). No observed female-female dispersal dyads included more than one river crossing whereas 12% of male-male dispersers crossed two rivers and 1% even all three rivers (Fig. 2).

Our dataset included 84 individuals (36 males and 48 females) with multiple geographic sampling locations. Among these, 6 males (17%) and 3 females (6%) were found to have crossed rivers. Although males appeared more likely to cross rivers (odds ratio = 2.96, 95% CI: 0.58–19.70), this difference was not statistically significant (Fisher’s exact test, *p* = 0.16).

### Males disperse over longer distances than females (hypothesis 2)

As expected, dispersal distances between male-male, female-female, and male–female dyads differed significantly (ANOVA, F_2_ = 44.47, *p* < 0.001). Dispersal distances among male-male dyads ($\overline{x}$ = 56.4 km ± 45.0 SD) were, on average, more than twice those of female-female dyads ($\overline{x}$ = 22.8 km ± 23.9 SD), while male–female dyads showed intermediate values ($\overline{x}$ = 46.8 km ± 42.3 SD). A post hoc Tukey test indicated that all three groups differed significantly from one another (Supplementary Fig. S2).

### Dispersal direction in males is random, i.e. unaffected by rivers (hypothesis 3)

Approximately half of the male-male dispersals (48%) fell into the category ‘orthogonal to rivers’ (direction D1 as opposed to direction D2 ‘parallel to rivers’) which did not differ from the chance probability of 0.5 (χ^2^ = 0.10, *p* = 0.75), thereby supporting our third hypothesis.

### Females are less likely to disperse orthogonal to rivers (hypothesis 4)

As hypothesized, significantly fewer female-female dispersals (40%) occurred ‘orthogonal to rivers’ than could be expected by chance (χ^2^ = 9.28, *p* = 0.001).

The shapes of the distributions for all dispersal angles of female-female and male-male dyads were significantly different (Kolmogorov–Smirnov test, D = 0.15, *p* = 0.02). The difference became even larger if only long dispersals (> 25 km) were compared (which are more likely to present a choice of river crossing). While the shape of distribution for dispersal angles of male-male dyads remained the same, the frequency of dispersal angles ‘orthogonal to rivers’ markedly decreased in female-female dyads (Kolmogorov–Smirnov test, D = 0.25, *p* = 0.008; Supplementary Fig. S3).

### The effect of rivers on genetic population structure is stronger for females (hypothesis 5)

**Fig. 3:**
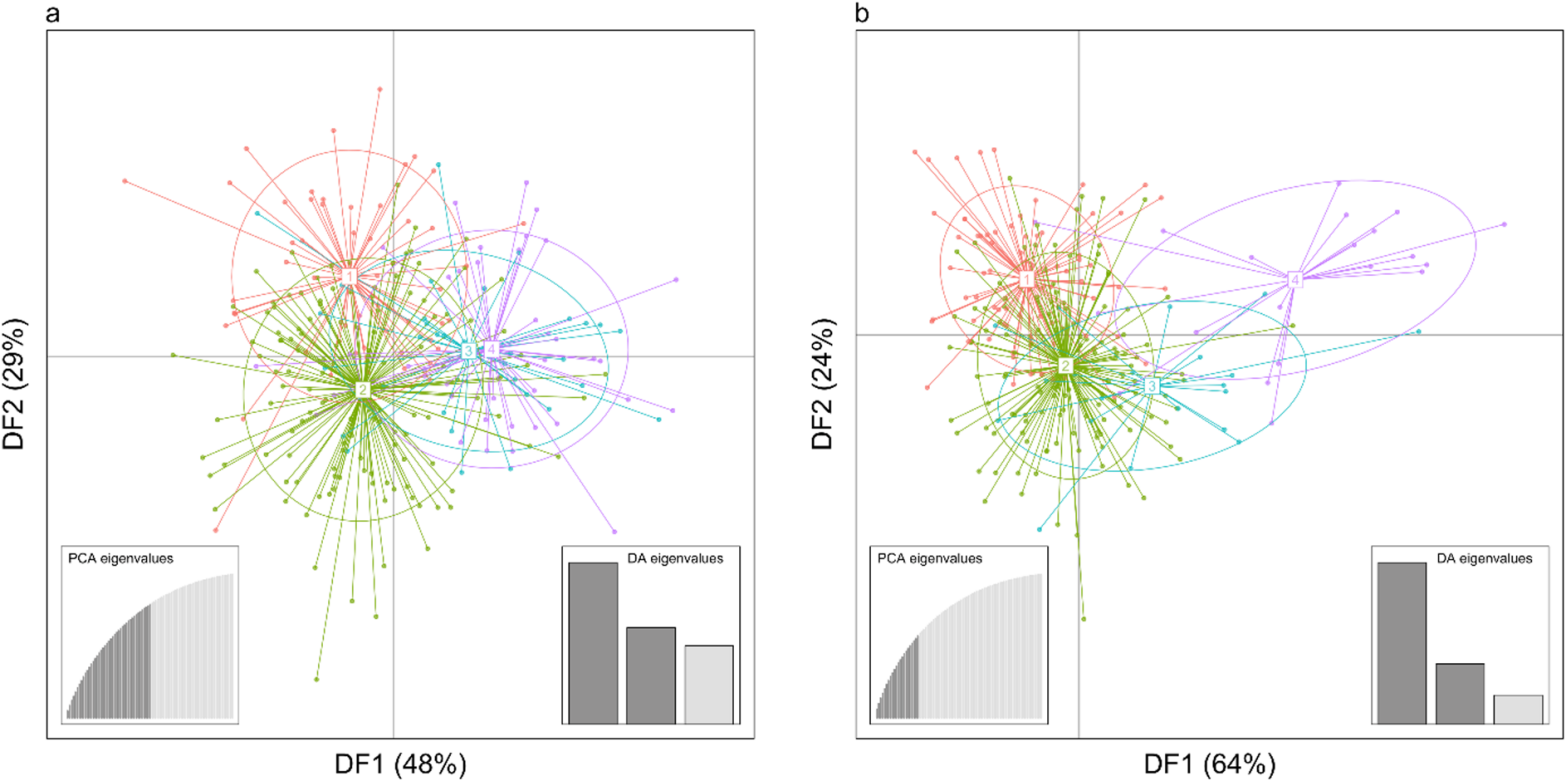
Discriminant Analysis of Principal Components (DAPC) scatterplot showing the genetic structure of 265 male (**a**) and 254 female (**b**) brown bears in Västerbotten County, Sweden. Dots represent individual SNP-based genotypes. Numbers and color correspond to the geographic group priors (four river-separated regions from south to north: 1—Ångerman, 2—Ume, 3—Vindeln, 4—North), shown together with their 95% inertia ellipses. The first two discriminant functions (DF1 and DF2) are shown on the axes and represent discriminant functions that maximize between-group genetic differentiation rather than total genetic variance.

We found only partial support for our fifth hypothesis. DAPC showed no clear genetic structuring corresponding to the four river-demarcated areas in Västerbotten for males and only weak structuring for females (Fig. 3). For females, the strongest genetic differentiation was shown for individuals from the northern region (cluster 4, Fig. 3b). Blind clustering (i.e., without a priori group assignment) weakly suggested two clusters for males and three to four clusters for females (Supplementary Fig. S4).

Visual interpretation of the bar and scree plots associated with the spatial PCA analyses suggested the presence of global (and absence of local) structure for both males and females with most of the spatially meaningful genetic variation being captured by the first PC axis (13.31% for males and 15.49% for females; Supplementary Fig. S5). Monte-Carlo tests for the presence of global and local structure confirmed the visual interpretation (Monte-Carlo test for global structure: males p_sim_ = 0.001, females p_sim_ = 0.001; Monte-Carlo test for local structure: males p_sim_ = 0.99, females p_sim_ = 0.99).

**Fig. 4:**
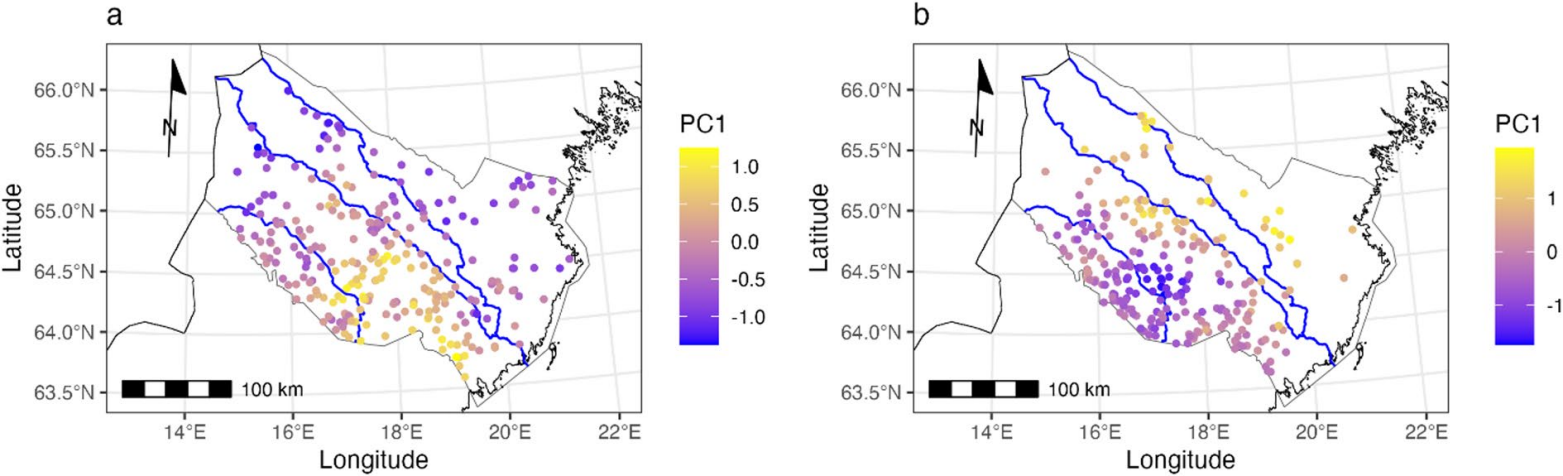
Results from a spatial PCA on genotypes of (**a**) male (n = 265) and (**b**) female (n = 254) brown bears plotted onto a map of Västerbotten County, Sweden. Tests indicated the presence of only global genetic structure, contained on the first PC axis. The dots represent individual genotypes with colors denoting the scores on PC1. Similarity in color corresponds to genetic similarity.

Males showed less spatial genetic structure in relation to river-demarcated regions than females. In females, genetic differentiation increased along a gradient from the southwest to the northeast (‘orthogonal to rivers’), but transitions did not exactly match the river boundaries (Fig. 4).

## Discussion

Rivers acted as a stronger barrier for dispersing females than for males, confirming a pattern previously only reported for American black bears^10,11^. Male-male dispersals were almost four times as likely to include river crossings than female-female dispersals and none of the detected dispersers in female-female dyads crossed more than one river whereas 13% of male-male dispersals included the crossing of two or three rivers. The smaller subset of individuals sampled at multiple locations showed a similar tendency, with males appearing nearly three times more likely to cross rivers than females (odds ratio = 2.96). However, this difference was not statistically significant, likely due to the small number of observed crossing events and the resulting wide confidence intervals (95% CI: 0.58–19.70).

These patterns can likely be explained by the same factors suggested for black bears. Male brown bears disperse farther than females and have larger home ranges^18^. This means that dispersing males are more likely to encounter and cross rivers in order to establish their territories. To do otherwise could lower individual fitness by reducing the likelihood of encountering unrelated, receptive females for mating^10^. Male bears also do not participate in rearing cubs and are thus unencumbered in their movements by dependent offspring. Females on the other hand are philopatric with home ranges often overlapping their maternal range^19^. This aligns with our findings of much shorter distances for female-female than male-male dispersals. However, simulated female dispersals (using random directions with distances drawn from the observed distance distribution) suggested that the lower frequency of river crossings by females is not simply an artifact of shorter dispersal distances as the frequency of river crossings in the simulation was significantly higher than the observed frequency of river crossings. Females also dispersed significantly less in directions orthogonal to rivers (i.e., perpendicular to the main river courses, where crossings would be required), whereas dispersal direction in males was random. This strongly suggests that these patterns are not simply a manifestation of spatial autocorrelation, but instead reveal behavioral differences in male and female dispersal patterns in relation to rivers.

As typical K-strategists^20^, female brown bears maximize their fitness by large investment into parental care. We therefore suspect that females may be deterred from river crossings due to the risk this would pose to their offspring, but could not specifically test this hypothesis.

Because rivers clearly did not impede the movement of male bears, we found no effect of rivers on the genetic structure of male bears in Västerbotten County. DAPC analysis did not show clear genetic separation between individuals sampled from within the four river-demarcated areas (Fig. 3a) nor did spatial PCA indicate genetic separation in relation to river boundaries (Fig. 4a).

For females our results suggested a weak effect of rivers on genetic structure. Females from the northernmost area did cluster more separately in the DAPC (Fig. 3b), supporting the notion that rivers act as barriers and that this also may be related to the characteristics of the river. While the rivers are similar in size (Supplementary Table S1), the Vindel River is the only unregulated of the three rivers and may therefore offer fewer crossing opportunities. In contrast, regulated rivers have dams that bears can use to cross, and directly below dams, periodically virtually dry riverbeds (Supplementary Fig. S6).

Spatial PCA showed increasing genetic differentiation among females in the direction orthogonal to rivers (i.e., from the southwest towards the northeast), but the three river courses did not demarcate clear boundaries (Fig. 4b). The latter is to be expected to some degree as all three rivers had female crossings, but no female crossed all three rivers (Fig. 2). We suspect that the genetic substructure in females is largely due to female philopatry, with rivers acting as natural obstacles that reinforce this pattern without fully preventing movement. Because males travel greater distances than females, nuclear markers are continuously recombined, preventing the development of strong genetic structure. Mitochondrial markers, on the other hand, may show some structuring, but this process would take much longer. Moreover, the relatively short time since glaciation, combined with occasional female dispersal across rivers, likely erodes any mitochondrial structuring faster than it can form.

Further research should address at which specific locations bears are crossing the rivers and if further sex-specific differences exist. For example, do river width, flow rate and river depth affect males and females differently and does the age of individuals play a role? Natal dispersal in bears is often strongest during spring, when mothers chase away their older offspring to focus on the younger cubs born during winter hibernation. Spring typically also coincides with the highest water levels and strongest currents in rivers due to snow melt, making especially the unregulated rivers risky to cross. In American black bears, Bond et al.^11^ showed that river depth and flow rate at crossing sites in Georgia were significantly lower than at random locations and White et al.^10^ reported that subadult males were more likely to cross the large Mississippi River than adult males or females of any age. This suggests that subadult males, possibly due to inexperience or a strong drive to establish territories, may be especially prone to risky behaviors during dispersal.

Bears in Sweden usually enter hibernation around the time of the first snowfall when ambient temperature reaches 0 °C^21^, which is well before the rivers freeze. In northern Sweden, exit from hibernation dens typically occurs from mid-April to May^22^. The demographic group to emerge earliest is adult males, followed by subadult individuals and females with cubs^22,23^. Ice break-up on rivers in the study area mostly occurs from March to April^24^. While early emerging male bears may still experience at least partially frozen rivers, it is unlikely that this accounts for the higher frequency of river crossings in males. Conditions during ice break-up and thinning ice during the period prior to break-up are likely to be perceived as even more dangerous than crossing ice-free rivers during the rest of the year.

Roads have also been implicated as barriers to brown bear movements^25,26^. For instance, Bischof et al.^25^ found that road networks constrained bear territories, with both males and females being less likely to be detected outside the network tile of their home range center, even after accounting for the effects of major rivers. Similarly, Skuban et al.^26^ demonstrated that the impact of roads on brown bear movements is closely tied to traffic volumes, with avoidance thresholds at 5000 vehicles per 24 h for males and 4000 vehicles per 24 h for females. At traffic volumes below 4000 vehicles per 24 h, no significant difference between the sexes in road-crossing behavior was observed.

In our study area, river valleys also contain roadways. However, the largest highway, the European route E12 flanking the Ume River, has an average traffic volume of less than 2000 vehicles per 24 h^27^. While roads in our study area may contribute to some resistance to bear movement, they are unlikely to fully explain the pronounced effect of sex on river crossings.

Our study highlights the need for further investigation into specific crossing sites and the factors driving sex-specific differences in river crossings, as well as the long-term effects of rivers as semipermeable barriers on the genetic structure of bear populations. Additionally, future research should explore whether this river effect interacts with other obstacles, such as roads, and examine these dynamics in other parts of Sweden. A clearer understanding of these processes could inform conservation strategies aimed at maintaining genetic connectivity in brown bear populations across fragmented landscapes.

## Methods

### Ethics statement

All research for this study was carried out in accordance with Swedish law and did not use experimental animals. We used a dataset containing 985 sexed SNP-based genotypes of brown bears from Västerbotten County in northern Sweden, which were derived from non-invasively collected fecal samples spanning the period from 2014 to 2019.

DNA was extracted using a QIAsymphony SP (Qiagen, Hilden, Germany) robot according to the manufacturer’s instructions. SNP genotyping was performed on a Fluidigm Biomark platform using a 96 SNP panel developed by Norman et al.^5^ for Scandinavian brown bears with modifications described in Norman and Spong^14^, which resulted in a final panel of four mtDNA SNPs for species confirmation, four Y-chromosome and three X-chromosome markers for sexing, and 85 autosomal SNPs characterizing individual genotypes. All statistical analyses were conducted in R version 4.4.2^17^, using a significance threshold of α = 0.05. Individuals were identified using the R-package *allelematch* (v2.5.3)^28^ with the maximum number of mismatching alleles tolerated when identifying individuals set at 12. For individuals that were represented by multiple samples, we calculated spatial center coordinates from the geographic coordinates of the samples using R-package *aspace* (v4.1.0)^29^. To determine relatedness between individuals, we used R-package *related* (v1.0)^30^ to calculate the Lynch-Ritland relatedness coefficient^31^, which has been shown to perform better than other relatedness estimators^32,33^ and used a threshold of r > 0.4 to assign first-order relatedness. We applied a threshold of 0.4 to accommodate natural variation in the relatedness coefficient (r), especially among full siblings. This threshold reduces the risk of misclassifying first order relatives with lower relatedness levels. The next highest level of relatedness, corresponding to second-order relatives (e.g., half-siblings), has an expected r of approximately 0.25, minimizing the likelihood of misclassification between first and second order relatives.

We chose three major rivers in Västerbotten (Ångerman River, Ume River, and Vindel River; see Supplementary Table S1 for details), which run from the mountains in the west to the coast in the east, to determine if they act as barriers for dispersal from south to north or vice versa. These rivers effectively divided the study area into four regions (Fig. 1). We also defined a line orthogonal to the rivers as the reference for dispersal angles (Fig. 1). For testing hypothesis 1 (males are more likely to cross rivers than females) we compared the proportions of dispersal events that included river crossings for both sexes using a Chi-square test with Yates’ continuity correction. Since female brown bears have been shown to disperse over shorter distances than males^18^, lower frequencies of river crossing during female-female dispersals could simply result from not encountering rivers rather than rivers constraining dispersal. To test this scenario, we simulated random female-female dispersals in the following manner: We first randomly drew (with replacement) 238 starting points (to match the observed 238 female-female dispersal dyads) from the pool of geographic coordinates of known female dispersers. To each starting point, we then randomly assigned a dispersal distance drawn (without replacement) from the distribution of observed dispersal distance and randomly assigned a dispersal direction between 0 and 360°. Thus, our simulated female-female dispersals matched the observed distances, but were random with regard to dispersal direction. We then determined the proportion of river crossings in the simulated data. We repeated this procedure 1,000 times and tested if the mean of the resulting distribution was greater than the observed proportion of river crossings using a one-sided t-test. In addition, we tested for differences in the proportion of river crossings between male and female individuals that were sampled multiple times at different locations using Fisher’s exact test.

To test hypothesis 2 (Dispersal distance differs between males and females), we calculated Euclidian distances for dispersals between female-female, male-male, and male–female pairs of first-order relatives and used ANOVA followed by Tukey’s post-hoc paired tests to test for differences in dispersal distances.

For testing hypotheses 3 and 4 (dispersal direction in males is unaffected by rivers and females are less likely to disperse orthogonal to rivers), we defined dispersal angles less or equal to 45° of the reference line (Fig. 1) as dispersal direction D1 (‘orthogonal to rivers’) and direction D2 (‘parallel to rivers’) for angles > 45° from the reference line. We then tested whether the probability of dispersing into direction D1 was significantly different from *p* = 0.5 (i.e., random) for males using a two-tailed one-proportion z-test and a one-tailed test to assess if females dispersed significantly less in direction D1 than could be expected by chance. Additionally, we used a two-sample Kolmogorov–Smirnov test to test for differences in the distribution of all dispersal angles for males and females for two scenarios; first, including all observed dispersal events and second, including only long dispersals (> 25 km), which are more likely to necessitate river crossings.

To investigate if the effect of rivers on genetic population structure is stronger for females than for males (hypothesis 5) we used Discriminant Analysis of Principal Pomponents (DAPC) and spatial Principal Component Analysis (sPCA) as implemented in the R-package *adegenet* (v2.1.10)^34,35^.

For DAPC, we defined individuals found in the four river-demarcated areas as prior groups. If landscape fragmentation by rivers affects genetic population structure, those spatially defined groups should correspond to genetic clusters in the DAPC, with stronger separation between clusters with increasing genetic differentiation between members of different clusters. We also used ‘blind’ k-means clustering (i.e., the male and female genotypes without any priori group memberships) to assess the numbers of clusters in the data (function *find.clusters()*). We evaluated the number of retained PCs for the DAPC using the a-score and via cross-validation methods^36^ and used the average of both methods for our analyses.

Spatial PCA yields scores summarizing both the genetic variability and the spatial structure among populations^34^. Global patterns of genotypes correspond to positive spatial autocorrelation, which is typically observed when populations are located along clines or split into groups (i.e., neighboring individuals are genetically more similar than random pairs) whereas local structures display negative autocorrelation, i.e. neighbors are genetically more distinct than would be expected from chance^34^. For the sPCA analysis we used the Delaunay triangulation which seeks to maximize the angles within the triangles of the connection network^37^. Since sPCA does not allow for duplicate coordinates in the data, we added a small amount of random noise (jitter) to the spatial coordinates of individuals. We assessed the presence of global and local genetic structure by first visually interpreting barplots and scree plots of the eigenvalues of the sPCA (Supplementary Fig. S5). Additionally, we also ran Monte-Carlo based tests for the presence of global and local structure in our data as implemented in *adegenet* (functions *global.rtest()* and *local.rtest()*, respectively).

## Supplementary Information

Below is the link to the electronic supplementary material.

Supplementary Material 1

## Data Availability

The data supporting this study are publicly available in the Dryad repository at 10.5061/dryad.zkh1893qr.
