# Supplementary material for: Rivers constrain female but not male dispersal and genetic structure in brown bears
Source: Sci Rep. 2026 Feb 9;16:5581. doi: 10.1038/s41598-026-38870-4 (PMC12891673; doi:10.1038/s41598-026-38870-4)
Supplement: Supplementary file 1 — Supplementary Material 1 [file 41598_2026_38870_MOESM1_ESM.pdf]

Supporting Information

Landscape and sex interaction in brown bear

TABLES

- Tab. S1 River profiles for the main three rivers in Västerbotten County, Sweden.

FIGURES

- Fig. S1 Histogram of the proportion of river crossings in simulated female-female brown bear dispersals.
- Fig. S2 Boxplot of dispersal distances for dyads of female, male, and male-female pairs of 1st order relatives for brown bears in Västerbotten County, Sweden.
- Fig. S3 Density distribution for the dispersal angles of male-male and female-female dyads of 1st order related brown bears in Västerbotten County, Sweden.
- Fig. S4 Results of the *find.clusters()* function in R-package *adegenet*
- Fig. S5 Barplots and scree plots of sPCA eigenvalues for male and female brown bears in Västerbotten County, Sweden.
- Fig. S6 Example pictures of a regulated and an unregulated river in the study area.

Table S1 River profiles for the three main rivers in Västerbotten county, Sweden. Mean and maximum flow rate refer to water volume at the river's mouth to the sea. The Vindel River is a tributary to the Ume River, thus flow rates at the mouth are only given for the latter. Source: The Swedish Meteorological and Hydrological Institute (SMHI, Faktablad 44-2010, available at: https://www.smhi.se)

**Name**	**Length (km)**	**Width (m)***	**Mean flow rate (m^3/s)**	**Max. flow rate (m^3/s)**	**Type**
Ångerman River	447	556 (591)	500	1330	regulated
Ume River	449	718 (912)	443	1365	regulated
Vindel River	450	271 (290)	-	-	unregulated

* Average width with standard deviation in parentheses. River widths were not available at SMHI. We therefore measured widths at 20 random locations along each river course (with a minimum distance of 5 km between measurements) using the Satellite layer in Google Maps.

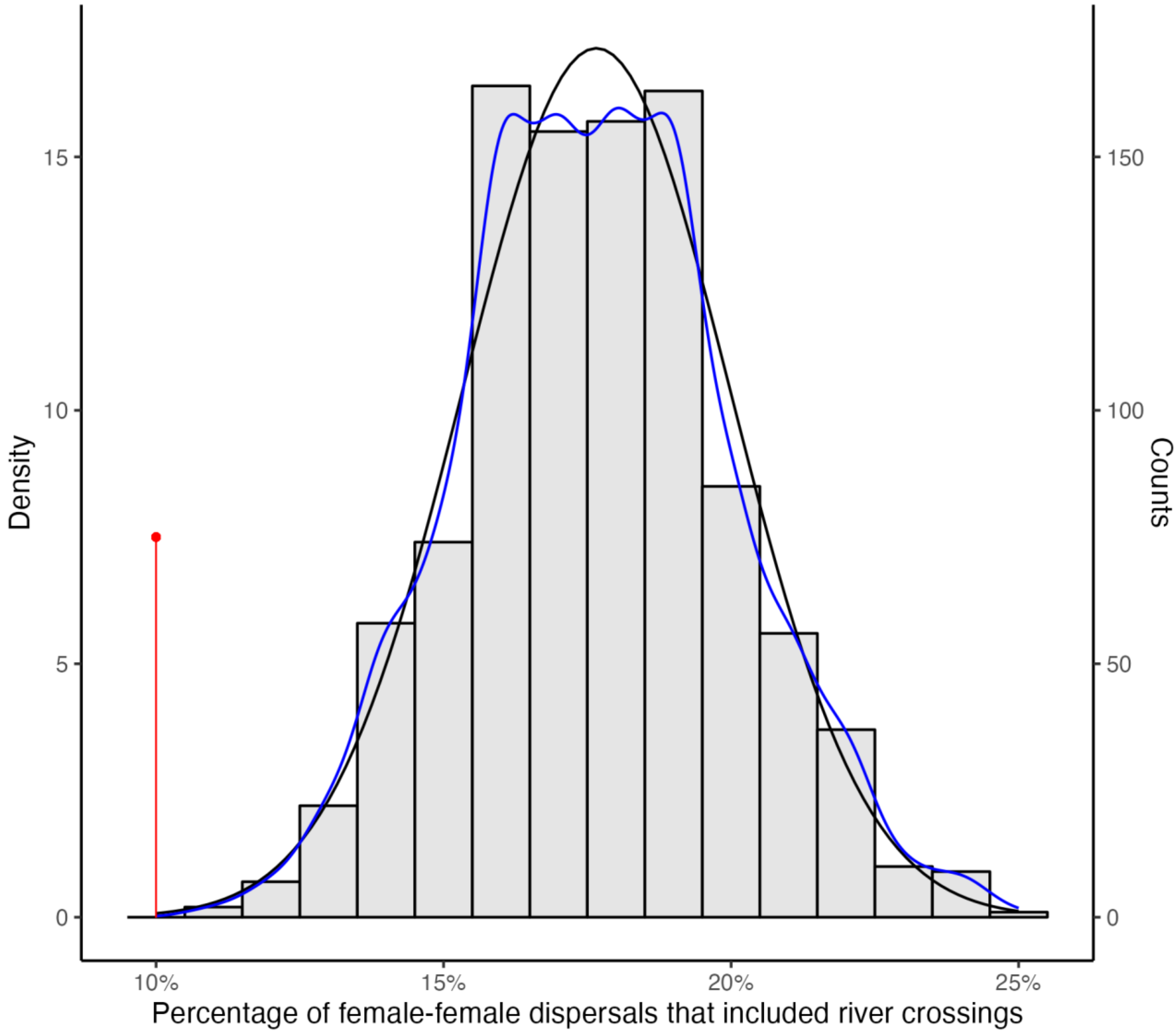

Fig. S1 Histogram of the proportion of river crossings in simulated female-female brown bear dispersals (N = 238 in each of 1,000 bootstrap iterations) in random directions but following the same distance distribution as observed dispersals. The observed proportion of river crossings (11%, indicated in red) is lower than the mean of the simulation results ($\bar{x}$ = 17.7 %) and fell outside the 97.5th percentile point (13 %) of the lower tail of the distribution. Fitted to the histogram are a normal distribution (in black) and the density distribution of the simulation results (in blue).

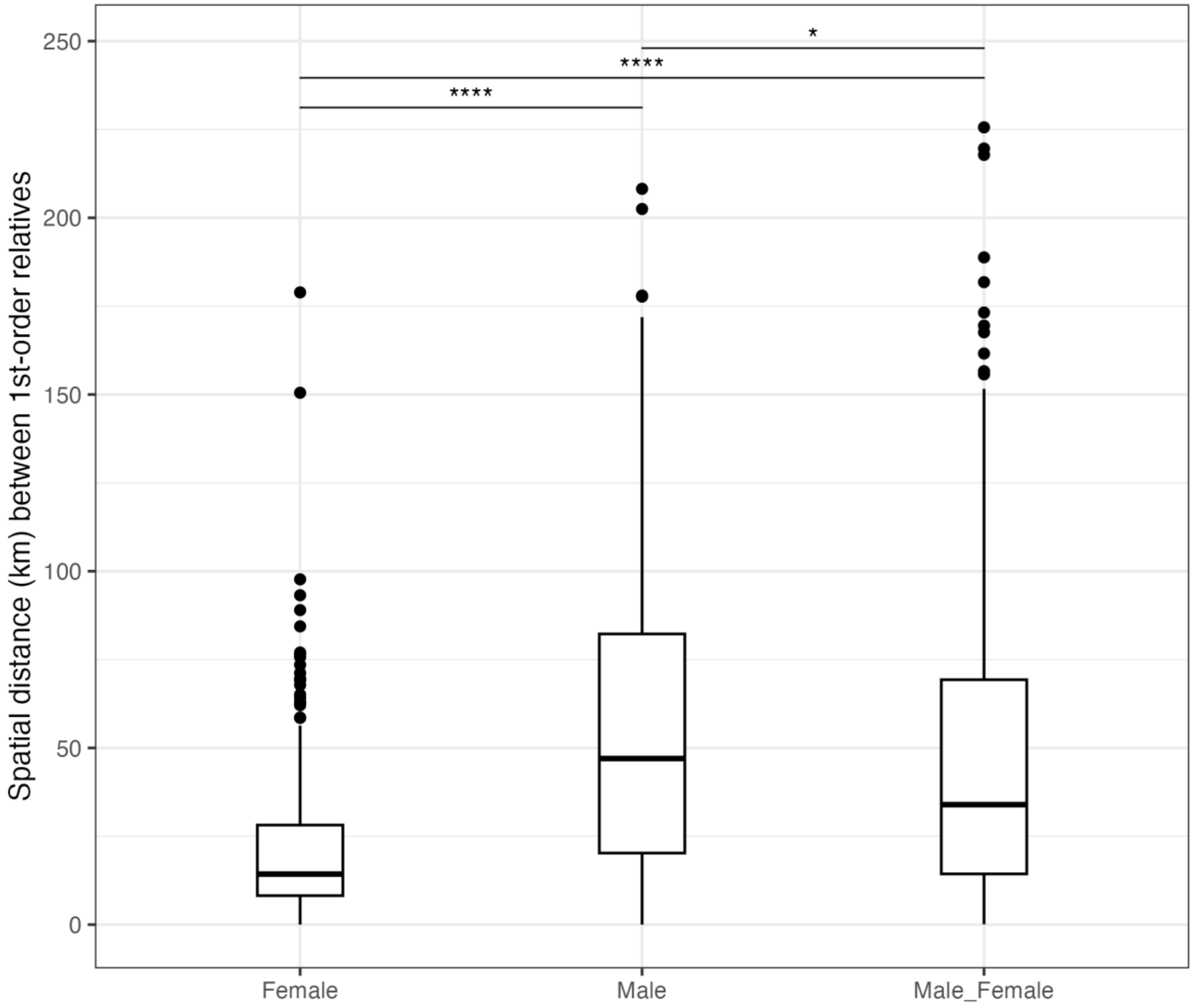

Fig. S2 Boxplot of dispersal distances for dyads of female, male, and male-female pairs of 1st order relatives for brown bears in Västerbotten County, Sweden. Significant differences between group means are indicated by asterisks (* $p < 0.05$, **** $p < 0.0001$) and based on post hoc Tukey-tests.

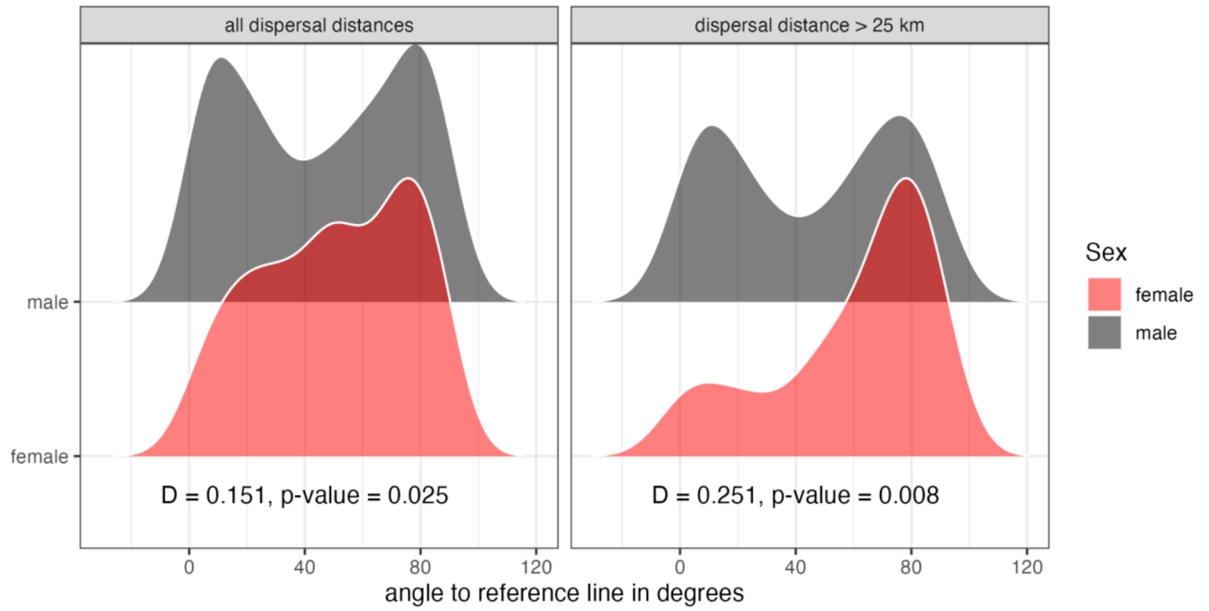

Fig. S3 Density distribution for the dispersal angles of male-male (gray) and female-female (red) dyads of first-order related brown bears in Västerbotten County, Sweden. Shown are the results for all distances (left facet) and long dispersals (> 25 km, right facet) together with the results of Kolmogorov-Smirnov tests for differences in the shape of distributions. Dispersal angles in the range 0 - 45° correspond to dispersals 'orthogonal to rivers') whereas angles > 45° represent dispersals 'parallel to rivers'.

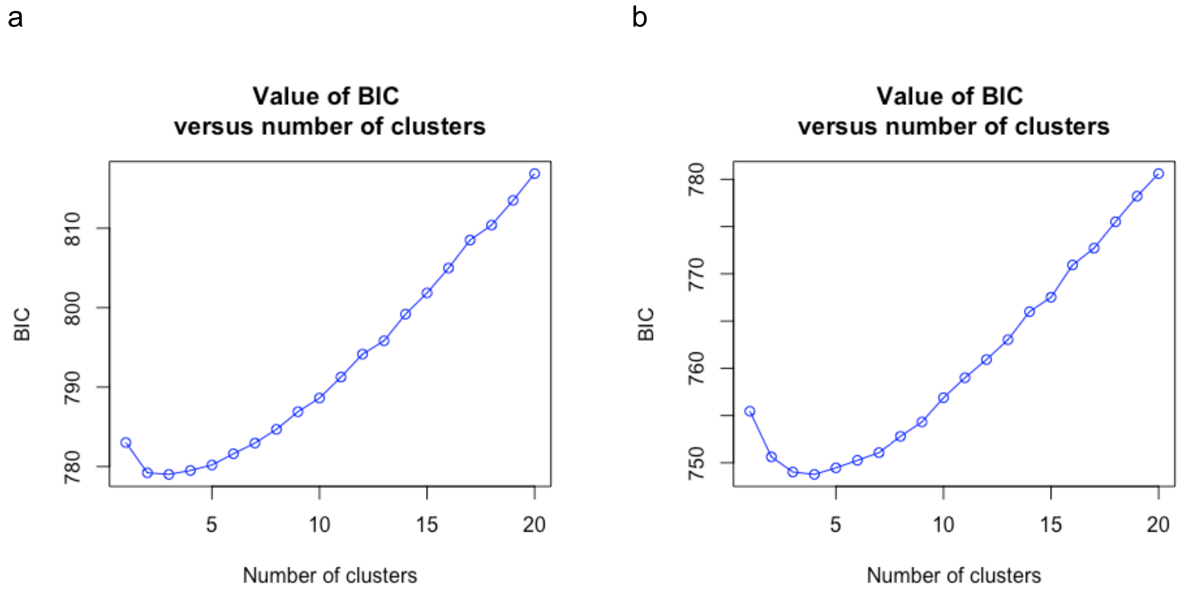

Fig. S4 Results of the *find.clusters()* function in R-package *adegenet* showing Bayesian information criterion (BIC) values for increasing values of k (= number of clusters), suggesting (a) one or two clusters for male and (b) three to four clusters for female brown bears in Västerbotten County, Sweden.

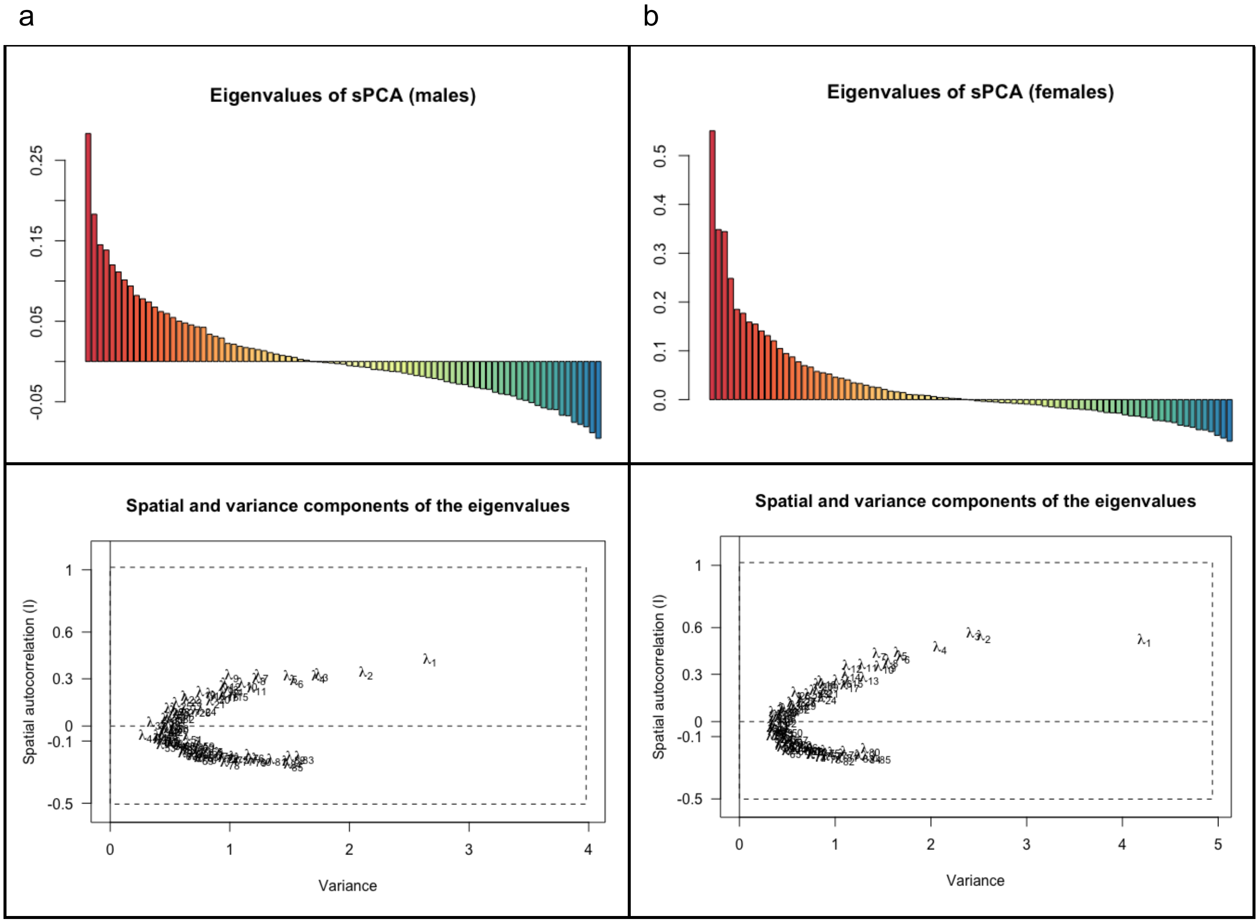

Fig. S5 Barplots (upper panel) and scree plots (lower panel) of sPCA eigenvalues for male (a) and female (b) brown bears in Västerbotten County, Sweden. In the bar plots, the presence of either global structure (reddish colors) or local structure (blueish colors) is indicated by more extreme eigenvalues. In the eigenvalue decomposition (scree) plots, sPCA eigenvalues are denoted as λ_i (with λ_1 corresponding to the highest positive eigenvalue).

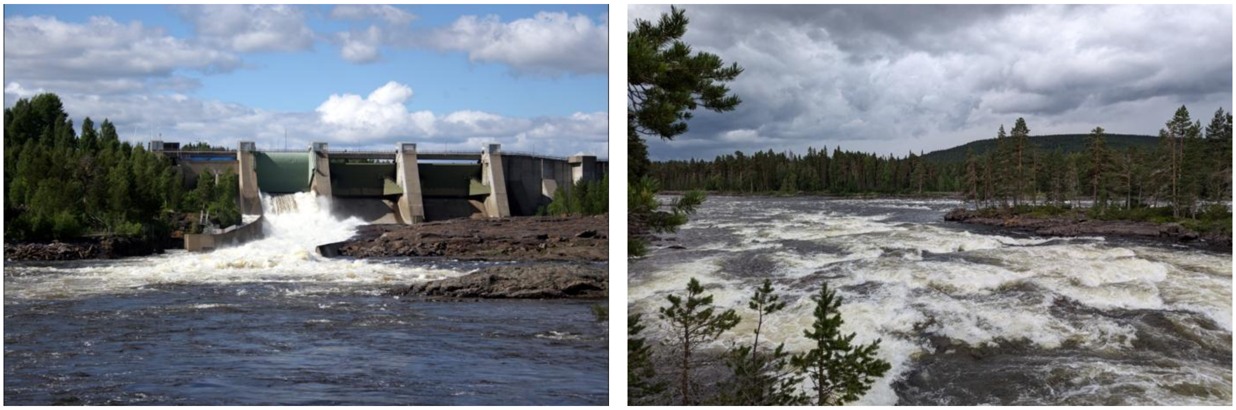

Fig. S6 Dams and periodically low water levels below dams may make regulated rivers like the Ume River (a) easier for bears to cross than unregulated rivers like the Vindel River (b).
